# Arbuscular Mycorrhizal Fungi Improve Tolerance of the Medicinal Plant *Eclipta prostrata* (L.) and Induce Major Changes in Polyphenol Profiles Under Salt Stresses

**DOI:** 10.3389/fpls.2020.612299

**Published:** 2021-01-15

**Authors:** Nguyen Hong Duc, Au Trung Vo, Imane Haddidi, Hussein Daood, Katalin Posta

**Affiliations:** ^1^Department of Genetics, Microbiology and Biotechnology, Szent István University, Gödöllõ, Hungary; ^2^Regional Knowledge Center, Szent István University, Gödöllõ, Hungary

**Keywords:** arbuscular mycorrhizal fungi, *Eclipta prostrata*, medicinal plant, phenolic compounds, bioactive compounds, secondary metabolites, salinity, salt tolerance

## Abstract

*Eclipta prostrata* (L.) is an important and well-known medicinal plant due to its valuable bioactive compounds. Microorganisms, including arbuscular mycorrhizal fungi (AMF), and salinity could directly impact plant metabolome, thus influencing their secondary metabolites and the efficacy of herbal medicine. In this study, the role of different single AMF species (*Funneliformis mosseae*, *Septoglomus deserticola*, *Acaulospora lacunosa*) and a mixture of six AMF species in plant growth and physio-biochemical characteristics of *E. prostrata* under non-saline conditions was investigated. Next, the most suitable AM treatment was chosen to examine the impact of AMF on physio-biochemical features and polyphenol profiles of *E. prostrata* under saline conditions (100 and 200 mM NaCl). The findings indicated that AMF mixture application resulted in more effective promotion on the aboveground part of non-saline plants than single AMF species. AM mixture application improved growth and salt tolerance of *E. prostrata* through increasing the activity of catalase, peroxidase (at 4 weeks), proline, and total phenolic content (at 8 weeks). Such benefits were not observed under high salinity, except for a higher total phenolic concentration in mycorrhizal plants at 8 weeks. Through high-performance liquid chromatography, 14 individual phenolic compounds were analyzed, with wedelolactone and/or 4,5-dicaffeoylquinic acid abundant in all treatments. Salinity and mycorrhizal inoculation sharply altered the polyphenol profiles of *E. prostrata*. Moderate salinity boosted phenolic compound production in non-AM plants at 4 weeks, while at 8 weeks, the decline in the content of phenolic compounds occurred in uncolonized plants subjected to both saline conditions. Mycorrhization augmented polyphenol concentration and yield under non-saline and saline conditions, depending on the growth stages and salt stress severity. Plant age influenced polyphenol profiles with usually a higher content of phenolic compounds in older plants and changed the production of individual polyphenols of both non-AM and AM plants under non-stress and salt stress conditions. A better understanding of factors (involving mycorrhiza and salinity) affecting the phenolic compounds of *E. prostrata* facilitates the optimization of individual polyphenol production in this medicinal plant.

## Introduction

*Eclipta prostrata* (L.), belonging to a family of medicinal plants (Asteraceae), is a native plant of Asia but is widely distributed in subtropical, tropical, and warm temperate regions on the globe (Liu et al., 2012). It is an important medicinal plant, which has been used in conventional systems of medicine and also by traditional healers, particularly in China, Japan, India, Vietnam, and other regions in the cure for various diseases (Yu et al., 2020). In fact, this medicinal plant has been utilized for the medication of respiratory diseases such as asthma, diphtheria, pertussis, and tuberculosis; as anti-HIV 1; and for diabetes type II; loose teeth; graying of hair; dizziness; hemoptysis; antihyperlipidemic, antihyperglycemic, and antioxidant activities (Chung et al., 2017; Feng et al., 2019; Yu et al., 2020); and mitigating the cognitive impairment induced by scopolamine (Jung et al., 2016). The plant possesses an outstanding therapeutic and medicinal value due to its valuable chemical composition such as alkaloids; alkenynes; cardiac glycosides; coumarins; flavonoids; flavones, e.g., luteolin; lipids; polyacetylene compounds; essentials oil; steroids; saponins; phytosterol; β-amyrin; triterpenes, e.g., ecalbatin and echinocystic acid; and coumestans, e.g., wedelolactone (Gani and Devi, 2015). Wedelolactone has been shown to have potent hepatoprotective or antihepatotoxicity, anti-inflammatory, and antitumor effects and neutralization of myotoxic and lethal activities of snake venom (Chung et al., 2017). Phenolic compounds, for instance, flavonoids, phenolic acids, and tannins extracted from this plant, have various biological activities, including anti-inflammatory, anti-atherosclerotic, and anticarcinogenic (Chung et al., 1998; Soobrattee et al., 2005). Noticeably, the environment and other factors such as harvest time, storage time, and geographical sources may influence the chemical components in *E. prostrata* (Chung et al., 2017).

Salinization is a growing problem in agricultural ecosystems, which jeopardizes plant growth and productivity. Salinity causes ion toxicity (Na^+^ and Cl^–^), nutritional imbalance, pigment degradation and inhibition of photosynthesis, oxidative and osmotic stress, limited leaf diffusion (flux) of CO_2_, modification of metabolic pathways, cell deformation, premature senescence, and ultimately cell death in the plant (Zelm et al., 2020). It is predicted that around one billion hectares traversing more than 100 countries face salt problems, and salinization is rapidly increasing with an estimated annual addition of 0.3–1.5 million hectares of farmland (Food and Agriculture Organization [FAO], 2015). Therefore, efficient strategies to cope with soil salinity under agricultural management systems could include salt-tolerant varieties and biotechnological approaches such as applying beneficial microbes capable of improving plant tolerance.

Arbuscular mycorrhizal fungi (AMF), one of the prevalent soil microbes, can colonize most terrestrial plant species’ roots. These symbiotic fungi have been reported to considerably offer various benefits to their host plants, such as enhanced uptake of mineral nutrients and water and increased tolerance to stressful environments (Baum et al., 2015). Remarkably, arbuscular mycorrhizal (AM) fungi could improve host plant tolerance to salinity stress by an array of physiological and biochemical mechanisms, including higher water-use efficiency, photosynthetic capacity, ionic homeostasis maintenance, osmoprotection, cell ultrastructure preservation, and strengthened antioxidant metabolism (Evelin et al., 2019). In lettuce, AM inoculation elevated proline accumulation and the activities of antioxidants such as catalase (CAT), superoxide dismutase (SOD), and ascorbate peroxidase, but lowered phenolic compound synthesis and oxidative stress with high growth in plants exposed to salt stress (Santander et al., 2019). Recently, AMF have been demonstrated to increase growth, nutrient acquisition (P, K^+^, Mg^2+^), and the ratios of total chlorophyll:carotenoids, Ca^2+^:Na^+^, Mg^2+^:Na^+^, and K^+^:Na^+^ in the shoots of the medicinal plant *Valeriana officinalis* under salinity stress (Amanifar and Toghranegar, 2020). The authors also showed a higher stimulated root proline, total soluble sugars, and total phenolics in the shoots of mycorrhizal plants subjected to saline conditions.

Bioactive compounds accumulated in medicinal plants are susceptible to changes in the growing seasons, growth years, and environmental factors (Li et al., 2020). Indeed, abiotic stresses are robust elicitors of secondary metabolite production in plants since they use their energy in defense mechanisms by activating specific biosynthesis pathways (Caretto et al., 2015; Toscano et al., 2019). Despite the deleterious effects of salinity, this abiotic stress is one of the major factors influencing the physiology, biochemistry, and synthesis of bioactive compounds in many herbs (Bistgani et al., 2019; Behdad et al., 2020; Boughalleb et al., 2020). Bistgani et al. (2019) illustrated that total phenolic content in *Thymus vulgaris* (L.) and *T. daenensis* Celak was inclined by approximately 20% after treatment with 60 mM NaCl, while leaf flavonoid content was enhanced by 38.6 and 36.6% in plants in response to 60 and 90 mM NaCl, respectively. Cinnamic acid, the main constituent in both plant species, was elevated by 31.4% in *T. vulgaris* in the presence of 60 mM NaCl (Bistgani et al., 2019). In a recent work, total flavonoid compounds, phenolic acids, and phenolic compounds in *Polygonum equisetiforme* plants were elevated with salinity levels remarkably at 300 mM NaCl (Boughalleb et al., 2020). An augmentation in total phenolic acids resulted mainly from an incline in gallic, protocatechuic, and quinic acids, followed by quercetin-3-*O*-galactoside, catechin, and epicatechin (Boughalleb et al., 2020).

On the other hand, the potential of AM inoculation to boost the biosynthesis of bioactive compounds such as phenols, alkaloids, and terpenes has been described in numerous medicinal plants; thus, fungal symbiosis could inflict significant changes in the pharmacological properties of medicinal plants (Kapoor et al., 2017). Previously, we reported that mycorrhizal inoculation modified the content of some polyphenols such as protocatechuic, 4-*O*-caffeoylquinic acid, 4,5-dicaffeoylquinic acid, luteolin, and quercetin-3-arabinoside of *E. prostrata* cultivated under different substrates with various nutrient supplies (Vo et al., 2019). In rosemary, mycorrhization altered polyphenolic profile distribution in the leaves. Four substances possessing strong antioxidant properties, namely, asiatic acid, ferulic acid, vanillin, and carnosol, were linked to rosemary plants treated with *Rhizoglomus irregulare* (Seró et al., 2019). Amanifar and Toghranegar (2020) pointed out that moderate salt stress stimulated a higher valerenic acid production in the medicinal plant *V. officinalis* L., which was more profound in the plants colonized by *Funneliformis mosseae*. Intriguingly, the potential of AMF, in combination with salinity, has not been fully noticed yet (Kapoor et al., 2017). AM application for the cultivation of medicinal plants in saline soils could be used to address the growing demand of medicinal plants for the pharmaceutical industry (Amanifar and Toghranegar, 2020). Moreover, it may offer the possibility of re-utilizing salt-affected soils under agricultural systems.

It is noteworthy that the synergistic effects of salt stress with other environmental factors on bioactive compounds in medicinal plants have been poorly understood (Isah, 2019). To the best of our knowledge, no studies on the interactive effects of salinity stress and AM inoculation on physio-biochemical parameters and polyphenol profiles of *E. prostrata* have been reported yet. Different AMF species or isolates could differently influence host plant growth and responses to stresses (Lee et al., 2013; Duc et al., 2018). Thus, this study aimed to explore the role of single AMF species and a mixture, including six AMF species on plant growth and physio-biochemical characteristics of *E. prostrata* under non-saline conditions. Next, the most suitable AM treatment was chosen to examine the impact of AMF on physio-biochemical features and polyphenol profiles of *E. prostrata* during plant growth and under salinity stresses. In practice, *E. prostrata* plants are usually cultivated and harvested before flowering; however, there is no information available to select the right harvest time to optimize individual bioactive compounds for pharmaceutical and cosmetic industries. Our hypotheses were as follows: (1) that mycorrhizal colonization could improve plant growth and tolerance to salt stresses, particularly moderate salinity, and (2) that AMF and salinity had interactive effects on the polyphenol profiles of this medicinal plant, which depended on plant age.

## Materials and Methods

### Plant Material, Arbuscular Mycorrhizal Preparation, and Inoculation

Seeds of *E. prostrata* (L.) from Hong Dai Viet Ltd (Vietnam) were used in our experiments. The mycorrhizal commercial inoculant Symbivit containing six AMF species, *Claroideoglomus etunicatum*, *Rhizoglomus microaggregatum*, *Rhizophagus intraradices*, *Claroideoglomus claroideum*, *Funneliformis mosseae*, and *Funneliformis geosporum*, was provided by Symbiom Ltd., Czechia. *Septoglomus deserticola* BEG 73 and *Acaulospora lacunose* BEG 78 were obtained from the International Bank for the Glomeromycota. *F. mosseae* SZIE originated from the collection of Szent István University. Three single AMF species were separately propagated by using *Medicago truncatula* and *Zea mays* as host plants cultured in autoclaved sand for 6 months. A mixture of spores, mycelia, infected root fragments, and sand from cultures was harvested for mycorrhizal inoculation. The inoculation dosage was 15 g of inocula per pot with about 2,400 infective propagules evaluated by the most probable number test (Porter, 1979). Mycorrhizal inocula were applied before transferring germinated seeds to pots of the experiments. In the preliminary experiment, control treatment representing pots without AMF inoculation was prepared in the following way. 15 g of autoclaved combined inoculum including *F. mossea*, *A. lacunose*, *Symbivit, S. deserticola* (each inoculum accounted for one-fourth of the amount) and 3 ml of a filtrate (<20 μm) of this combined inoculum to provide a microbial population with non-AM propagules. In the salt stress experiment, 15 g of sterilized Symbivit and 3 ml of a filtrated solution of Symbivit were used for pots without mycorrhizal inoculation.

### Plant Growth and Experiment Design

#### Preliminary Experiment: Impact of Different Single AMF Species and a Mixture of AM Inoculation on *E. prostrata* Plant Performance Under Non-stress Conditions

The seeds were sterilized with NaOCl 1%, then washed with distilled water several times and put on a filter paper in Petri dishes at 26°C for germination for 3 days. Germinated seeds were placed in 0.5-L plastic pots filled with an autoclaved mixture of sand and peat (60:40%) (v/v). The chemical properties of the sand:peat substrate have pH 6.9, N (%) 0.6%, P 681.29 mg kg^–1^, K 2,819 mg kg^–1^, carbonate (%) 17.18%, and dry matter content (m/m%) 54% (Vo et al., 2019). The AM inocula were placed adjacent to seedling roots. An experiment setup according to a randomized complete block design included five different treatments: (1) plants inoculated with *S. deserticola*, (2) plants inoculated with *A. lacunose*, (3) plants inoculated with *F. mosseae*, (4) plants inoculated with a mixture of six AMF species (Symbivit), and (5) plants without AM inoculation (control). Each treatment had 12 biological replicates, equivalent to 12 pots (one plant per pot). Therefore, five treatments (four different kinds of AM treatment and control) with 12 replicates resulted in a total of 60 pots. Pots were put in a climatic chamber EKOCHL 1500 (24/28°C, 60% relative humidity, 16/8 h photoperiod, light intensity 600 μmol m^–2^ s^–1^) and watered once a week. At 4 and 8 weeks of growth, plants were harvested for measurements. Root colonization, plant height, fresh root and shoot weight, leaf number, and leaf area were examined. Fully expanded leaves (excluding petioles) were immediately frozen in liquid nitrogen and stored at −80°C until total phenolics and proline content determination.

#### Salt Stress Experiment: Impact of AM Inoculation and Salinity Stress on Plant Performance and Polyphenol Profiles of *E. prostrata*

Based on the preliminary experiment results, the mixture of six AMF species (Symbivit) was chosen for AM treatment in this experiment. A factorial experiment was performed using a randomized complete block design with two factors: (1) salinity levels (0, 100, and 200 mM NaCl) (Chauhan and Johnson, 2008) and (2) mycorrhizal inoculation (inoculated with either the mixture of six AMF species or the sterilized AM inoculant as control). After surface-disinfected seeds were germinated, they were sown in each plastic pot (10 × 6 × 14 cm in size) containing 3 kg of sterilized sand and peat (60:40%) (v/v) substrate. Each treatment had 10 biological replicates; therefore, six treatments (3 salinity levels × 2 mycorrhizal inoculations) with 10 replicates resulted in a total of 60 pots (one plant per pot). Pots were put in a climatic chamber EKOCHL 1500 (24/28°C, 60% relative humidity, 16/8 h photoperiod, light intensity 600 μmol m^–2^ s^–1^). During plant growth (8 weeks), non-stress plants were watered with 100 ml of tapping water per pot once a week, while salt stress treatments were applied by watering with 100 ml of 100 or 200 mM NaCl for each pot once a week. Fresh shoot and root weight, plant height, leaf number, leaf area, stem diameter, chlorophyll fluorescence, and mycorrhizal colonization rate were examined at 4 and 8 weeks of growth. The fully expanded leaves (excluding petioles) were immediately frozen in liquid nitrogen and stored at -80°C until analysis of proline, superoxide dismutase, peroxidase, catalase, and polyphenol components.

### Assessment of Arbuscular Mycorrhizal Colonization

Five plants per treatment were randomly selected, then their roots were washed to remove the substrate and cleared with 10% KOH for 10 min, acidified using 2% HCl and 0.05% Trypan blue in 1:1:1 water:glycerin:lactic acid overnight. Thirty root fragments (1 cm long) were mounted on a glass slide, and four glass slides per plant were examined according to Trouvelot et al. (1986) using the MYCOCALC software in the preliminary experiment. In the salt stress experiment, 60 root fragments per technical replicate and four technical replicates per plant were used to evaluate mycorrhizal colonization according to the gridline intersect method (Giovanetti and Mosse, 1980).

### Leaf Area and Chlorophyll Fluorescence Measurement

Leaf area was determined according to the method of Glozer (2008). The maximum quantum efficiency of photosystem II photochemistry (*F*_*v*_/*F*_*m*_), a chlorophyll fluorescence parameter, was measured after 30 min of dark adaptation using a Walz-PAM 2500 (Germany) fluorometer according to the method of Oxborough and Baker (1997). The measurements were implemented on the fourth leaf from a single plant’s shoot apex in each treatment with five biological replicates.

### Proline Content Determination

Proline content was quantified by the acid ninhydrin procedure of Bates et al. (1973). A half gram of leaf samples from each treatment was homogenized in 10 ml of 3% aqueous sulfosalicylic acid. Afterward, it was centrifuged at 10,000 rpm for 15 min. Two milliliters of the supernatant, 2 ml of glacial acetic acid, and ml ninhydrin acid were blended, then incubated at 100°C for 1 h. The reaction was terminated in an ice bath; subsequently, the chromophore was extracted with 4 ml toluene. Its absorbance at 520 nm was measured by U-2900 UV-VIS spectrophotometer (Hitachi). Proline concentration (μmol proline per g of fresh weight) was estimated from the standard curve.

### Measurement of Total Phenolic Content

Total phenolic concentration was measured by the Folin–Ciocalteu assay (Lister and Wilson, 2001). Briefly, 2 g of leaves were blended well with 20 ml of 60% ethanol and subsequently filtered. One milliliter of filtrate and 0.5 ml of Folin–Denis reagent were transferred to a tube, then mixed completely. Next, 1 ml of saturated Na_2_CO_3_ was added after 3 min at room temperature. The mixture was completed to 10 ml with distilled water and incubated for 30 min at room temperature. The absorbance at 760 nm was recorded, and total phenolics content was presented as mg gallic acid per g fresh weight.

### Measurement of Antioxidant Enzymatic Activities

Frozen leaves (0.5 g) were homogenized in 3 ml of 50 mM Tris–HCl buffer (pH 7.8) containing 1 mM Na_2_EDTA and 7.5% (w/v) polyvinylpyrrolidone K25 and centrifuged at 10,000 × *g* at 4°C for 20 min. The supernatants were used for peroxidase, superoxide dismutase, and catalase assays. The protein content of all leaf extracts was estimated by the method of Bradford (1976).

*Peroxidase* (POD, EC 1.11.1.7) activity was measured by the method of Rathmell and Sequeira (1974). Shortly, the reaction mixture (2.2 ml) containing 0.1 M sodium phosphate buffer (pH 6.0), 100 μl of 12 mM H_2_O_2_, and 100 μl of 50 mM guaiacol with 10 μl of plant extract was used to measure the POD activity at 436 nm in 5 min. The enzyme activity was expressed as the changes in absorbance mg^–1^ protein min^–1^.

*Superoxide dismutase* (SOD, EC 1.15.1.1) activity was determined spectrophotometrically at 560 nm following by the method of Beyer and Fridovich (1987). Briefly, the reaction mixture (2 ml) consisted 50 mM phosphate buffer (pH 7.8), 2 mM EDTA, 0.025% Triton X-100, 55 μM Nitroblue tetrazolium (NBT), 9.9 mM L-methionine, 20 μl of crude extract, and 20 μl of 1 mM riboflavin. The absorbance was read at 560 nm. One unit of SOD activity (U) was defined as the required enzyme volume to lead to 50% inhibition of the NBT decline under the assay conditions.

*Catalase* (CAT, EC 1.11.1.6) activity was measured by the method of Aebi (1984). The reaction mixture consisted of 1 ml of 10 mM of hydrogen peroxide and 2 ml of 50 mM potassium phosphate buffer (pH 7.0) and 20 μl leaf extract. The absorbance decrease at 240 nm of the reaction was recorded as the deposition level of H_2_O_2_. The enzyme activity was presented as the changes in absorbance mg^–1^ protein min^–1^.

### HPLC Determination of Polyphenols

From each well-homogenized aerial part of fresh material of *E. prostrata*, a 0.5-g sample was taken and crushed in a crucible mortar with quartz sand. Twenty milliliters of a mixture of 44% EtOH, 4% MeOH, 10% water, and 2% acetic acid was gradually added with crushing and then transferred to a 100-ml Erlenmeyer flask. The macerate was subjected to an ultrasonication force using an ultrasonic water bath device (Model USD-150, Raypa) for 4 min, followed by mechanical shaking (GLF3005) for 15 min. The mixture was kept overnight at 4°C and filtered through Albet-DF400125 type filter paper. Before injection onto the HPLC column, it was further cleaned up by passing through a 0.22-mm PTFE HPLC syringe filter. Nucleosil C18-100, 3 μm, 240 × 4.6 mm Protect-1 HPLC column (Macherey-Nagel, Duren, Germany) was used to separate phenolic compounds using a gradient elution of 1% formic acid in water (A) and acetonitrile (B) with a flow rate of 0.6 ml min^–1^. Gradient elution began with 2% B, changed to 13, 25, and 40% B in 10, 5, and 15 min, respectively, and finally turned to 2% B in 5 min. The HPLC determination was performed using a Hitachi Chromaster HPLC with a Model 5160 pump, a Model 5260 autosampler, a Model 5310 column oven, and a Model 5430 diode-array detector. The separation and data processing were operated by OpenLab CDS software. The peaks were identified by comparing their retention times and spectral characteristics with available standards such as quercetin-3-arabinoside, luteolin-glucoside, luteolin-7-*O*-glucoside, luteolin, wedelolactone, demethyl wedelolactone, caffeic acid, 3,4-*O*-dicaffeoylquinic acid, 3,5-dicaffeoylquinic acid, 4-*O*-caffeoylquinic acid, 4,5-dicaffeoylquinic acid, 5-*O*-caffeoylquinic acid, ferulic acid, and feruloylquinic acid (Sigma-Aldrich Ltd., Hungary). For the quantification of phenolic compounds, each peak area was integrated at the maximum absorption wavelength, and the concentrations were calculated by relating the areas of the peaks to those of the available external standards (Merken and Beecher, 2000). The standard materials were singly injected as external standards and chromatographed with the samples as well.

### Statistical Analysis

Statistical analysis was implemented using the SAS 9.1 (SAS Institute, Cary, NC, United States) package for Windows. In the preliminary experiment, differences in plant growth traits, mycorrhizal colonization, proline, and total phenolics among AMF treatments were analyzed by one-way analysis of variance and Tukey *post hoc* tests. In the salinity stress experiment, two-way analysis of variance (GLM procedure in SAS) was applied with explanatory variables (factors) of AM inoculation and salt stress levels as well as their interaction. Moreover, Tukey *post hoc* tests were applied. In addition, two-sample, two-tailed *t*-test was applied using MS Excel to compare each particular treatment result at 4 and 8 weeks. PCA was carried out by the XLSTAT program to identify patterns, i.e., interactions among the studied variables and treatments, in polyphenolic data of *E. prostrata* with and without AMF under different salinity stress levels including no salinity stress.

## Results

### Inoculation of the AM Mixture Improved Aboveground Biomass and Total Phenolic Content of *E. prostrata* Under Non-stress Conditions (the Preliminary Experiment)

#### Mycorrhizal Colonization and Plant Growth Parameters

Microscopic observation of the roots showed that no AM colonization in non-AM plants (control plants) was detected. In contrast, plants in mycorrhizal treatments were successfully colonized by three single AMF species and the mixed AM inoculant. The colonization rate of plants infected by *A. lacunose* was lowest (22.9%) and significantly lower than plants treated with other single AMF species and the mixture of AMF (from 47.8 to 54.1%) 4 weeks after inoculation. Interestingly, no significant differences in this rate among 8-week plants treated with different mycorrhizal inoculants (ranged from 49.5 to 59.5%) were found (Table 1).

**TABLE 1:** Growth parameters of *Eclipta prostrata* inoculated with different arbuscular mycorrhizal fungi or not inoculated under non-saline conditions 4 and 8 weeks after inoculation (the preliminary experiment).

Treatment	Fresh root weight (g plant^–1^)		Fresh shoot weight (g plant^–1^)		Leaf number (leaf plant^–1^)		Plant height (cm plant^–1^)		Leaf area (cm^2^ plant^–1^)		Mycorrhizal colonization (%)	
						
	4 weeks	8 weeks	4 weeks	8 weeks	4 weeks	8 weeks	4 weeks	8 weeks	4 weeks	8 weeks	4 weeks	8 weeks
No AMF	1.49 ± 1.05^a^	1.81 ± 0.13^a^	4.44 ± 1.12^ab^	6.01 ± 0.91^b^	14.89 ± 4.8^a^	20 ± 5.53^a^	15.38 ± 3.07^a^	52.71 ± 7.41^a^	15.18 ± 2.73^a^	17.48 ± 0.91^ab^	0	0
*F. mosseae*	1.67 ± 0.19^a^	2.38 ± 0.62^a^	3.26 ± 0.30^bc^	6.05 ± 1.16^b^	12.89 ± 5.1^a^	20.86 ± 5.8^a^	9.44 ± 2.28^b^	40.88 ± 10.05^ab^	14.87 ± 2.00^ab^	16.38 ± 5.43^b^	54.1 ± 4.2^a^	59.5 ± 4.7^a^
*S. deserticola*	1.69 ± 0.40^a^	2.02 ± 0.18^a^	2.09 ± 0.17^*c*^	3.15 ± 0.55^c^	13.78 ± 4.29^a^	18.86 ± 4.14^a^	8.51 ± 1.78^b^	34 ± 7.57^b^	11.22 ± 0.94^b^	12.80 ± 2.62^b^	47.8 ± 6.6^a^	53.1 ± 5.8^a^
*A. lacunose*	1.83 ± 0.76^a^	2.33 ± 0.16^a^	3.07 ± 0.90^bc^	4.98 ± 1.20^bc^	17 ± 6.68^a^	22.85 ± 11.36^a^	10.72 ± 4.36^b^	38.71 ± 3.60^b^	14.79 ± 1.67^ab^	16.83 ± 1.22^b^	22.9 ± 3.6^b^	49.5 ± 9.2^a^
Symbivit	1.53 ± 0.03^a^	2.37 ± 0.30^a^	4.95 ± 0.30^a^	10.58 ± 1.86^a^	12.89 ± 2.47^a^	21.43 ± 4.86^a^	11.64 ± 1.57^ab^	46 ± 5.62^ab^	16.58 ± 1.37^a^	24.36 ± 1.79^a^	50.5 ± 5.4^a^	56.3 ± 6.8^a^

*The means ± standard deviation are shown (*n* = 5 for mycorrhizal colonization, *n* = 4 for other parameters). Different letters in each column indicate significant difference according to the Tukey test (*P* < 0.05) among treatments 4 and 8 weeks after inoculation. AMF, arbuscular mycorrhizal fungi.*

Regarding plant growth parameters, there were no significant differences in fresh root weight and leaf number among all treatments at 4 and 8 weeks (Table 1). Treatment with the mixture of six AMF dramatically increased fresh shoot weight by 76% in relation to non-AM plants after 8 weeks of growth. By contrast, plants inoculated with *S. deserticola* displayed a substantial decline in fresh shoot weight by 52.9 and 47.5% after 4 and 8 weeks of plant growth, respectively, in comparison with that of the corresponding non-mycorrhizal plants. Notably, inoculation of *F. mosseae*, *S. deserticola*, and *A. lacunose* considerably reduced plant height compared with uncolonized plants at 4 weeks, while the decline did not occur in the mixed AM treatment at both stages of plant growth. In terms of leaf area, no beneficial effects of different AM inoculations were observed in host plants.

#### Proline and Total Phenolic Concentration

**FIGURE 1:**
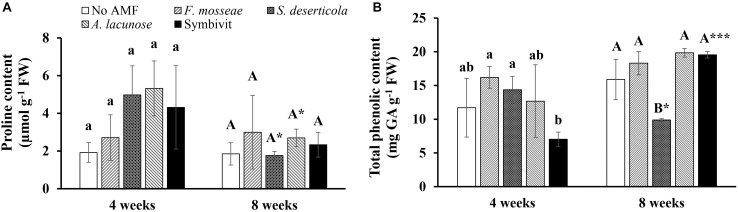
Proline **(A)** and total phenolic content **(B)** of *Eclipta prostrata* inoculated with *Funneliformis mosseae*, *Septoglomus deserticola*, *Acaulospora lacunose*, and Symbivit or not inoculated 4 and 8 weeks after inoculation (the preliminary experiment). AMF, arbuscular mycorrhizal fungi. Each bar shows the mean ± standard deviation (*n* = 3). Different regular and capital letters indicate significant differences among treatments according to the Tukey test (*P* < 0.05) 4 and 8 weeks, respectively, after inoculation. *, *** Indicate significant difference at *P* < 0.05 and *P* < 0.001, respectively, according to the two-tailed *t*-test for the same treatments between 4 and 8 weeks after inoculation.

Under non-stress conditions, inoculation with different species of AMF did not induce substantially higher proline concentrations in plants compared with non-AM plants during plant growth (Figure 1A). Remarkable decreases in the proline content were recorded in plants inoculated with *S. deserticola* (by 64.6%) and *A. lacunose* (49.3%) at the later stage compared with plants at the early stage of plant growth (*P* < 0.05). Similarly, there were no significant differences in total phenolic content between control plants and mycorrhizal plants after 4 weeks of growth (Figure 1B). Nonetheless, the treatment of Symbivit remarkably reduced the total phenolic concentration in plants by 56.8 and 51% relative to plants inoculated with *F. mosseae* and *S. deserticola*, respectively. At 8 weeks, the content of total phenolics in plants treated by the mixed inoculant was dramatically enhanced by 178.5% versus that at 4 weeks (*P* < 0.001). No significant differences in total phenolic content among control plants and plants treated with *A. lacunose* or Symbivit were detected 8 weeks after inoculation. Conversely, the total phenolic level in *S. deserticola* colonized plants at 8 weeks considerably declined by 31.2% (*P* < 0.05) in relation to that at 4 weeks. Moreover, *S. deserticola* caused a sharp reduction in total phenolic concentration in plants, as compared with non-AM plants (by 37.8%) and plants colonized by other fungal symbionts (by 46% versus *F. mosseae*, 50.2% versus *A. lacunose*, and 49.5% versus Symbivit) after 8 weeks of growth.

### Arbuscular Mycorrhizal Fungi Enhanced Plant Tolerance of *E. prostrata* to Moderate Salt Stress (the Salt Stress Experiment)

#### Root Colonization and Growth Parameters

**FIGURE 2:**
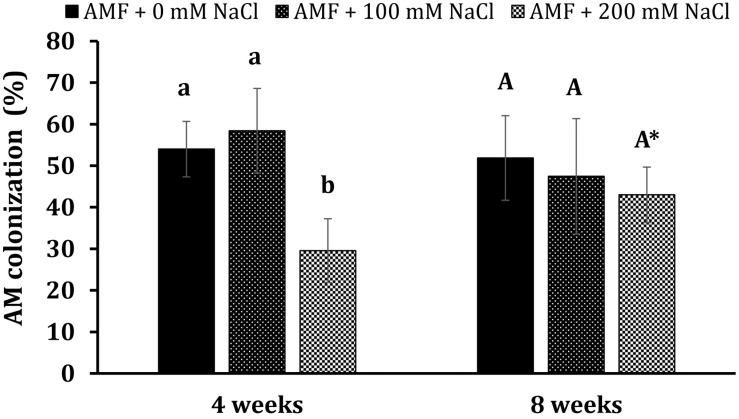
Arbuscular mycorrhizal colonization in the roots of *Eclipta prostrata* plants under non-stress and moderate (100 mM NaCl) and high saline (200 mM NaCl) conditions 4 and 8 weeks after inoculation. * Indicates a significant difference at *P* < 0.05 according to the two-tailed *t*-test in the same treatments between 4 and 8 weeks after inoculation.

Non-AM plants had no mycorrhizal colonization during plant growth. After 4 weeks of growth, the mycorrhizal colonization rate of AM plants obtained 54% under non-stress conditions, while the rate was 58.4% in those treated with 100 mM NaCl (Figure 2). No significant differences could be found between mycorrhizal plants under non-stress conditions and salt stress at 100 mM NaCl. Nonetheless, high salinity (200 mM NaCl) considerably decreased the colonization percentage to 29.6% at this plant growth stage. Interestingly, we did not find any substantial differences in mycorrhizal colonization rates among colonized plants under non-stress and saline conditions at 8 weeks. Their rates were 51.9, 47.4, and 43% in mycorrhizal plants under non-stress and moderate and high salt stress. The percentage of AM colonization in AM plants under high saline conditions at the later stage was significantly elevated (*P* < 0.05) relative to those at the early stage.

Exposure of *E. prostrata* plants to salt stresses, particularly at 200 mM NaCl, led to a considerable decrement in most growth parameters tested at both plant growth stages (Table 2). Under non-stress conditions, mycorrhizal inoculation substantially enhanced fresh root weight (only 75.6% at 8 weeks), fresh shoot weight (101 and 125%), leaf number (89 and 107%), stem diameter (70 and 47.1%), and leaf area (81.5 and 99.6%) at 4 and 8 weeks as compared with those of non-AM plants, while plant height remained unchanged in AM plants. In the presence of 100 mM NaCl, increases in fresh shoot weight (93% at 8 weeks), leaf number (68.1 and 96.3% at 4 and 8 weeks, respectively), and leaf area (59.7 and 88.5%) at both times measured in colonized plants were observed, compared with those of non-AM plants. AM colonization also markedly elevated the leaf area at 4 weeks (by 101%) in plants treated with 200 mM NaCl in comparison with the corresponding uncolonized plants.

**TABLE 2:** Growth parameters of *Eclipta prostrata* inoculated with arbuscular mycorrhizal fungi or not inoculated under non-stress and moderate and high saline conditions 4 and 8 weeks after inoculation (the salt stress experiment).

Treatment		Fresh root weight (g plant^–1^)		Fresh shoot weight (g plant^–1^)		Leaf number (leaf plant^–1^)		Plant height (cm plant^–1^)		Stem diameter (mm plant^–1^)		Leaf area (cm^2^ plant^–1^)	
						
Stress conditions	Mycorrhizal inoculation	4 weeks	8 weeks	4 weeks	8 weeks	4 weeks	8 weeks	4 weeks	8 weeks	4 weeks	8 weeks	4 weeks	8 weeks
Non-stress	AMF-	1.17 ± 0.05^ab^	1.68 ± 0.56^b^	2.43 ± 0.42^b^	4.4 ± 0.7^b^	10.0 ± 1.5^c^	15.2 ± 1.5^bc^	11.3 ± 6.0^a^	24.6 ± 6.6^ab^	2.0 ± 0.3^b^	2.93 ± 0.2^b^	8.73 ± 0.7^b^	11.93 ± 2.8^bc^
	AMF +	1.68 ± 0.4^a^	2.95 ± 0.72^a^	4.02 ± 0.4^a^	9.9 ± 2.6^a^	18.9 ± 2.9^a^	31.5 ± 10.1^a^	13.7 ± 6.0^a^	25.5 ± 6.5^a^	3.4 ± 0.7^a^	4.31 ± 0.8^a^	15.85 ± 2.6^a^	23.81 ± 3.7^a^
100 mM NaCl	AMF-	0.53 ± 0.3^bcd^	0.15 ± 0.05^c^	0.99 ± 0.33^cd^	1.4 ± 0.1^c^	9.4 ± 0.4^c^	11.1 ± 1.5^c^	6.9 ± 2.2^a^	10.9 ± 3.0^c^	1.9 ± 0.1^b^	2.13 ± 0.1^b^	5.24 ± 0.8^*cd*^	7.63 ± 1.0^c^
	AMF +	0.78 ± 0.3^bc^	0.41 ± 0.06^c^	1.82 ± 0.6^bc^	2.7 ± 0.3^b^	15.8 ± 3.2^ab^	21.8 ± 3.7^b^	7.9 ± 2.1^a^	13.3 ± 2.2^bc^	2.4 ± 0.1^ab^	2.45 ± 0.1^b^	8.37 ± 0.6^b^	14.38 ± 3.7^b^
200 mM NaCl	AMF-	0.03 ± 0.00^d^	0.13 ± 0.02^c^	0.44 ± 0.1^d^	0.9 ± 0.4^c^	9.4 ± 0.5^c^	9.0 ± 1.7^c^	3.8 ± 0.5^a^	5.0 ± 0.6^c^	1.4 ± 0.2^b^	2.06 ± 0.4^b^	3.28 ± 0.6^d^	7.28 ± 1.5^c^
	AMF +	0.11 ± 0.02^cd^	0.19 ± 0.04^c^	0.66 ± 0.5^cd^	1.6 ± 0.6^bc^	12.8 ± 1.1^bc^	11.7 ± 0.3^c^	5.6 ± 0.7^a^	10.1 ± 2.1^c^	1.8 ± 0.3^b^	2.36 ± 0.4^b^	6.60 ± 0.5^bc^	9.15 ± 1.8^bc^

Source of variation (ns, not significant, *P ≤ 0.05, **P ≤ 0.01, ***P ≤ 0.001)													

Mycorrhizal inoculation (M)		*	*	***	***	***	***	ns	ns	**	**	***	***
Salt stress (S)		***	***	***	***	**	**	*	***	***	***	***	***
M × S		ns	*	ns	**	*	ns	ns	ns	ns	Ns	*	*

*The means ± standard deviation are presented (*n* = 5 for mycorrhizal colonization, *n* = 3 for other parameters). Different letters in each column indicates significant difference according to the Tukey test (*P* < 0.05) among treatments 4 and 8 weeks after inoculation. AMF, arbuscular mycorrhizal fungi; ns, non-significant difference.*

#### Chlorophyll Fluorescence

**FIGURE 3:**
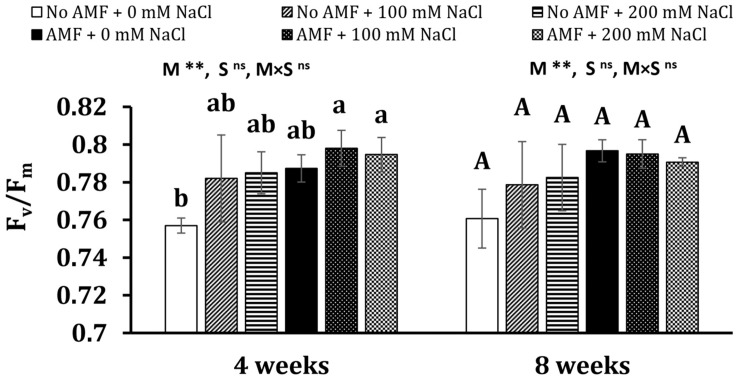
Maximal photochemical efficiency of photosystem II (*F*_*v*_/*F*_*m*_) in the leaves of *Eclipta prostrata* inoculated with arbuscular mycorrhizal fungi or not inoculated under non-stress and moderate (100 mM NaCl) and high saline (200 mM NaCl) conditions 4 and 8 weeks after inoculation. AMF, arbuscular mycorrhizal fungi. Each bar shows the mean ± standard deviation (*n* = 5). Different regular and capital letters indicate significant differences among treatments according to the Tukey test (*P* < 0.05) 4 and 8 weeks, respectively, after inoculation. **, significant differences at *P* < 0.01. ns, not significant. AMF, arbuscular mycorrhizal fungi. M, mycorrhizal inoculation effect. S, salt stress effect. M × S, the interaction between mycorrhizal inoculation and salt stress.

Although salt stresses slightly increased the maximal photochemical efficiency of photosystem II (*F*_*v*_/*F*_*m*_) in plants, no significant differences between mycorrhizal and non-mycorrhizal plants were found under the same conditions at 4 and 8 weeks of growth (Figure 3). Mycorrhizal treatment was the main factor substantially influencing *F*_*v*_/*F*_*m*_ at 4 and 8 weeks (*P* < 0.01), whereas the effect of salt stress was statistically significant on this parameter at 4 weeks (*P* < 0.05).

#### Proline Concentration

**FIGURE 4:**
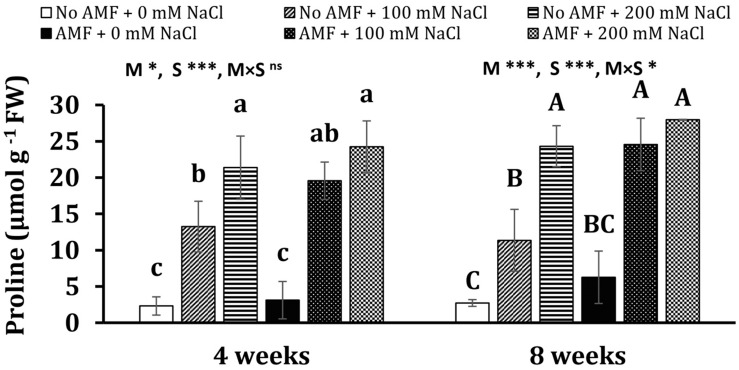
Proline concentration in the leaves of *Eclipta prostrata* inoculated with arbuscular mycorrhizal fungi or not inoculated under non-stress and moderate (100 mM NaCl) and high saline (200 mM NaCl) conditions 4 and 8 weeks after inoculation. Each bar shows the mean ± standard deviation (*n* = 3). Different regular and capital letters indicate significant differences among treatments according to the Tukey test (*P* < 0.05) 4 and 8 weeks, respectively, after inoculation. *, ***, significant differences at *P* < 0.05, 0.001. ns, not significant. AMF, arbuscular mycorrhizal fungi. M, mycorrhizal inoculation effect. S, salt stress effect. M × S, the interaction between mycorrhizal inoculation and salt stress.

Salinity heightened proline concentrations in mycorrhizal and non-mycorrhizal plants at 4 weeks (Figure 4). In detail, 4.7- and 8.2-folds of proline content in non-AM plants exposed to 100 and 200 mM NaCl over the control (non-AM plants) were detected, while 5.3- and 6.8-folds of proline level in AM plants under moderate and high saline conditions over non-stress mycorrhizal plants, respectively, were recorded. There are no significant differences between AM and non-AM plants under the same conditions (no stress and moderate and high salinity). A nearly similar trend was observed at 8 weeks of growth. Plants exposed to salt stresses substantially accumulated a higher proline content in comparison with non-exposed ones. Notably, under moderate salinity, the proline level in AM plants was 116% higher than non-AM plants. The effects of mycorrhizal inoculation (M) and salt stress (S) were statistically significant on proline concentration measured at 4 and 8 weeks (at least *P* < 0.05) with the existence of interaction between the two factors at 8 weeks (*P* < 0.05).

#### Antioxidant Enzymatic Activities

**FIGURE 5:**
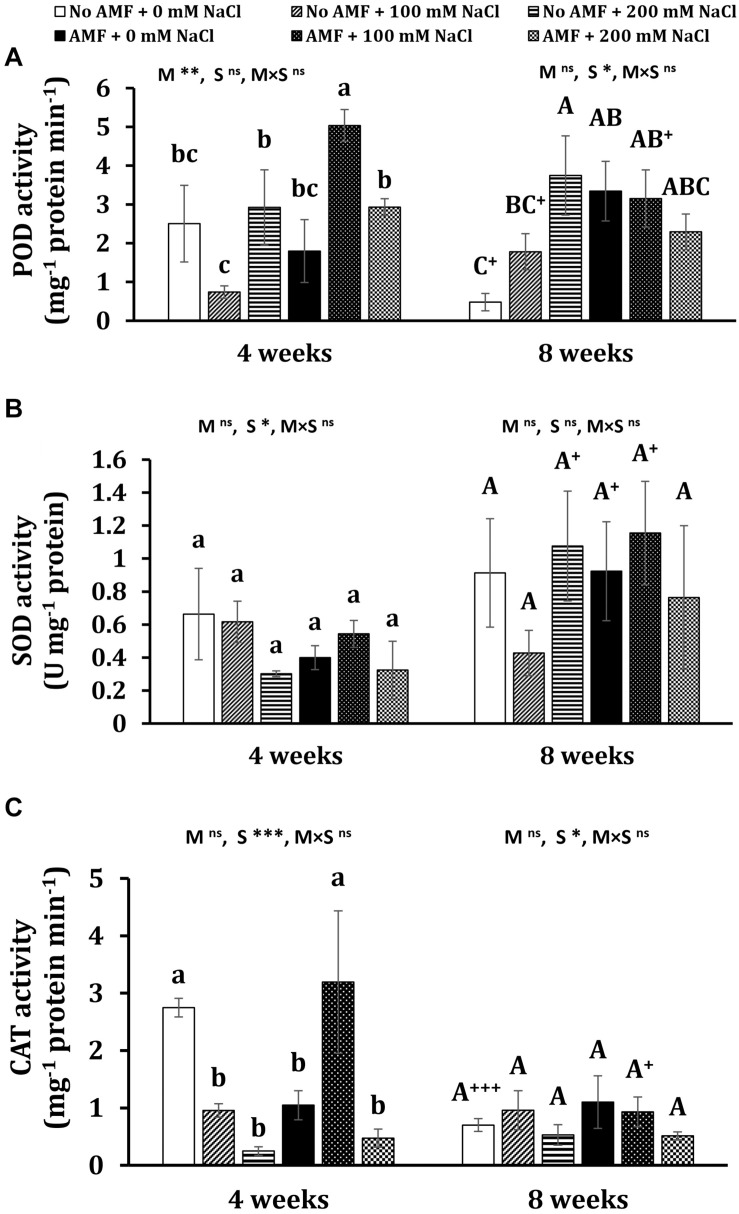
Peroxidase (POD) **(A)**, superoxidase dismutase (SOD) **(B)**, and catalase (CAT) **(C)** activity in the leaves of *Eclipta prostrata* inoculated with arbuscular mycorrhiza or not inoculated under non-stress and moderate (100 mM NaCl) and high saline (200 mM NaCl) conditions 4 and 8 weeks after inoculation. AMF, arbuscular mycorrhizal fungi. Each bar shows the mean ± standard deviation (*n* = 3). Different regular and capital letters indicate significant differences among treatments according to the Tukey test (*P* < 0.05) 4 and 8 weeks, respectively, after inoculation. +, ++, +++ indicate a significant difference between the same treatments 4 and 8 weeks after inoculation at *P* < 0.05, *P* < 0.01, and *P* < 0.001, respectively, according to the two-tailed test. ns, non-significant. *, **, ***, significant differences at *P* < 0.05, 0.01, 0.001. AMF, arbuscular mycorrhizal fungi. M, mycorrhizal inoculation effect. S, salt stress effect. M × S, the interaction between mycorrhizal inoculation and salt stress.

At the early stage of plant growth, mycorrhizal plants gained the highest POD activity under moderate salt stress, while the activity of this enzyme was lowest in non-AM plants subjected to 100 mM NaCl (Figure 5A). No significant differences could be seen in other treatments. At the later stage, POD activity was considerably lowered (by 80.8%, *P* < 0.05) in non-AM plants under non-stress conditions and (by 37.4%, *P* < 0.05) in moderate-salted mycorrhizal plants, but it substantially leaped (by 140%, *P* < 0.05) in uncolonized plants exposed to 100 mM NaCl. Salt treatments remarkably induced almost three- and seven-folds higher POD activity in non-stress uncolonized plants subjected to 100 and 200 mM NaCl, respectively, at 8 weeks. In contrast, both saline levels did not elevate POD activity in colonized plants. However, AM inoculation triggered an increase in POD activity by nearly sixfold in non-stress plants. Under moderate and high salt stress, no significant differences in POD activity were found between non-AM and AM plants. Mycorrhizal treatment markedly impacted POD at 4 weeks (*P* < 0.01), while salinity remarkably affected POD at 8 weeks (*P* < 0.05).

In terms of SOD activity, the observed differences between mycorrhizal and non-mycorrhizal plants under non-stress and salt levels were not statistically significant at 4 and 8 weeks (Figure 5B). There were substantial increments in this enzyme activity in non-AM plants under high salt stress (by 257%, *P* < 0.05), non-stress AM plants (by 131%, *P* < 0.05), and mycorrhizal plants exposed to moderate saline conditions (by 112%, *P* < 0.05) at the later stage versus the early stage of plant growth. Salinity considerably affected SOD at 4 weeks (*P* < 0.05).

Under non-stress conditions, mycorrhizal application significantly dropped CAT activity in plants at 4 weeks (Figure 5C). Moderate salt stress triggered a substantially higher level (by 205%) of this enzyme activity in colonized plants but remarkably lessened it (by 65.2%) in non-AM plants as compared with the corresponding ones. When plants were exposed to high salt concentration, no changes in CAT activity were recorded in mycorrhizal plants. Conversely, CAT activity was markedly reduced (by 90.9%) in uncolonized plants in comparison with those under non-stress conditions. No significant differences in CAT activity in both mycorrhizal and non-mycorrhizal plants under all conditions were found at 8 weeks of growth. Nevertheless, profound declines in CAT activity in non-AM plants under non-stress (by 292%, *P* < 0.001) and AM plants exposed to moderate salt stress (by 70.8%, *P* < 0.05) 8 versus 4 weeks after inoculation were observed. Salinity remarkably affected CAT at 4 (*P* < 0.001) and 8 weeks (*P* < 0.05).

### Arbuscular Mycorrhizal Fungi Altered Individual Phenolic Compounds of *E. prostrata* Under Non-saline and Saline Conditions (the Salt Stress Experiment)

**FIGURE 6:**
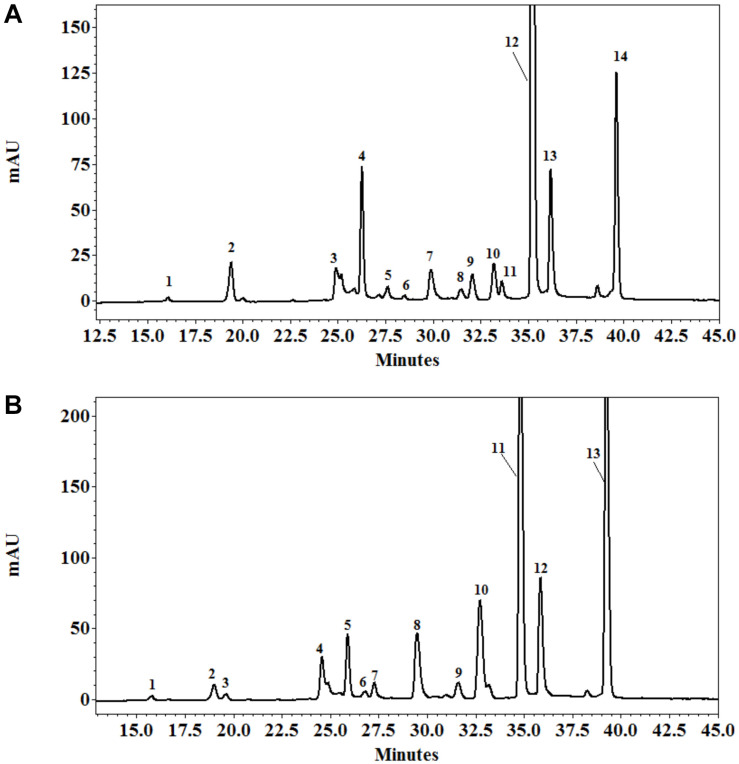
HPLC profile of polyphenols from the leaves of *Eclipta prostrata* separated on C18 Protect-1, 250 × 4.6 mm eluated with a gradient of acetonitrile in 1% formic acid solution 4 **(A)** and 8 weeks **(B)** after inoculation. Peak identifications of **(A)**: 1 = 5-*O*-caffeoylquinic acid; 2 = 4-*O*-caffeoylquinic acid; 3 = caffeic acid; 4 = 3,4-*O*-dicaffeoylquinic acid; 5 = 3,5-dicaffeoylquinic acid; 6 = luteolin-glucoside; 7 = luteolin-7-*O*-glucoside; 8 = ferulic acid; 9 = quercetin-3-arabinoside; 10 = demethyl wedelolactone; 11 = feruloylquinic acid; 12 = 4,5-*O*-dicaffeoylquinic acid; 13 = luteolin; 14 = wedelolactone. Peak identifications of **(B)**: 1 = 5-*O*-caffeoylquinic acid; 2 = 4-*O*-caffeoylquinic acid; 3 = caffeic acid; 4 = 3,4-*O*-dicaffeoylquinic acid; 5 = 3,5-dicaffeoylquinic acid; 6 = luteolin-glucoside; 7 = luteolin-7-*O*-glucoside; 8 = ferulic acid; 9 = quercetin-3-arabinoside; 10 = demethyl wedelolactone; 11 = 4,5-dicaffeoylquinic acid; 12 = luteolin; 13 = wedelolactone.

**FIGURE 7:**
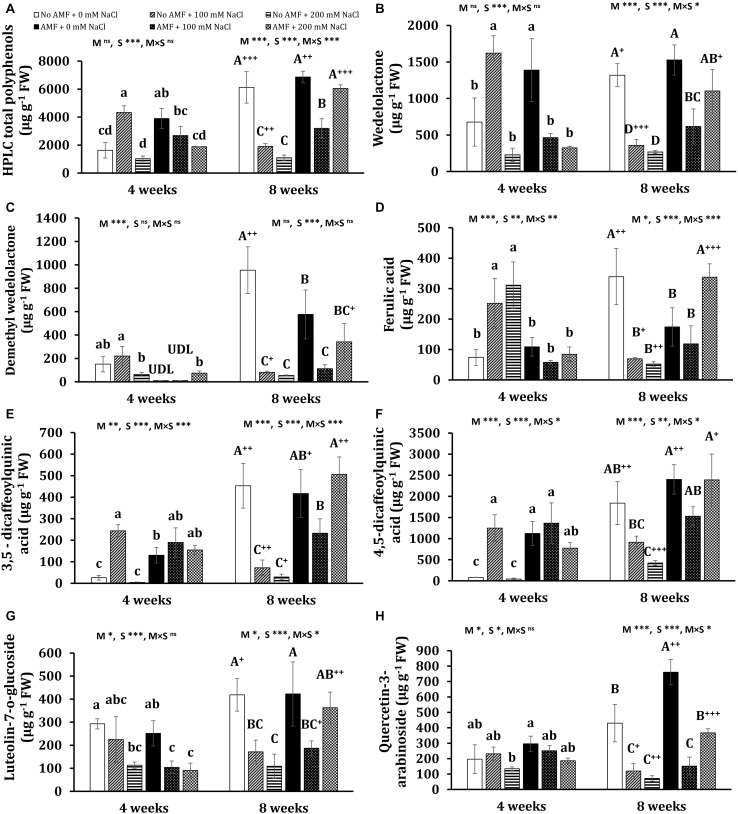
Contents of HPLC total polyphenols **(A)** and major polyphenols: wedelolactone **(B)**, demethyl wedelolactone **(C)**, ferulic acid **(D)**, 3,5-dicaffeoylquinic acid **(E)**, 4,5-dicaffeoylquinic acid **(F)**, luteolin-7-*O*-glucoside **(G)**, and quercetin-3-arabinoside **(H)** in the leaves of *Eclipta prostrata* inoculated with arbuscular mycorrhiza or not inoculated under non-stress and moderate (100 mM NaCl) and high saline (200 mM NaCl) conditions 4 and 8 weeks after inoculation. Each bar shows the mean ± standard deviation (*n* = 3). Different regular and capital letters indicate significant differences among treatments 4 and 8 weeks after inoculation, respectively, according to the Tukey test (*P* < 0.05). +, ++, +++ Indicate significant differences between the same treatments 4 and 8 weeks after inoculation at *P* < 0.05, *P* < 0.01, and *P* < 0.001, respectively, according to the two-tailed test. ns, non-significant. *, **, ***, significant differences at *P* < 0.05, 0.01, 0.001. AMF, arbuscular mycorrhizal fungi. M, mycorrhizal inoculation effect. S, salt stress effect. M × S, the interaction between mycorrhizal inoculation and salt stress. UDL, under the detection limit.

**FIGURE 8:**
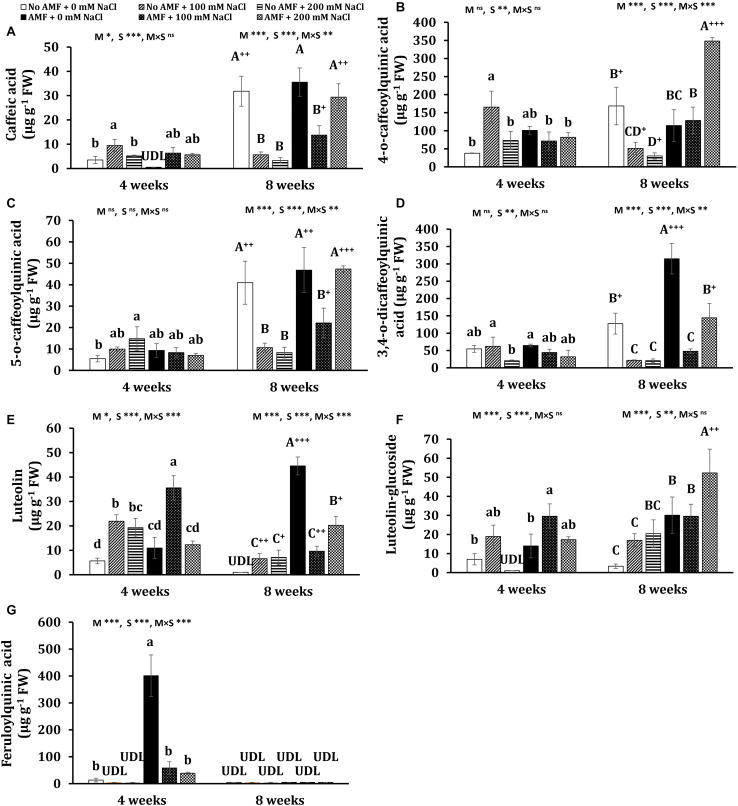
Contents of polyphenols: caffeic acid **(A)**, 4-*O*-caffeoylquinic acid **(B)**, 5-*O*-caffeoylquinic acid **(C)**, 3,4-*O*-dicaffeoylquinic acid **(D)**, luteolin **(E)**, luteolin-glucoside **(F)**, and feruloylquinic acid **(G)** in the leaves of *Eclipta prostrata* inoculated with arbuscular mycorrhiza or not inoculated under non-stress and moderate (100 mM NaCl) and high saline (200 mM NaCl) conditions 4 and 8 weeks after inoculation. Each bar shows the mean ± standard deviation (*n* = 3). Different regular and capital letters indicate significant differences among treatments 4 and 8 weeks after inoculation, respectively, according to the Tukey test (*P* < 0.05). +, ++, +++ Indicate significant differences between the same treatments 4 and 8 weeks after inoculation at *P* < 0.05, *P* < 0.01, and *P* < 0.001, respectively, according to the two-tailed test. ns, non-significant. *, **, ***, significant differences at *P* < 0.05, 0.01, 0.001. AMF, arbuscular mycorrhizal fungi. M, mycorrhizal inoculation effect. S, salt stress effect. M × S, the interaction between mycorrhizal inoculation and salt stress. UDL, under the detection limit.

The quantitative and qualitative measurements of polyphenols in the leaves of *E. prostrata* were implemented by HPLC-DAD analysis. The gradient elution applied was able to efficiently separate 14 phenolic constituents in plants 4 weeks after growth, namely eight hydroxycinnamates (caffeic acid, ferulic acid, 3,4-*O*-dicaffeoylquinic acid, 3,5-dicaffeoylquinic acid, 4-*O*-caffeoylquinic acid, 4,5-dicaffeoylquinic acid, 5-*O*-caffeoylquinic acid, feruloylquinic acid), four flavonoids (luteolin-glucoside, luteolin, luteolin-7-*O*-glucoside, quercetin-3-arabinoside), and two coumarins (wedelolactone and demethyl wedelolactone) (Figure 6A), whereas only 13 components of polyphenols (feruloylquinic acid was under detection limit) were determined in 8-week plants (Figure 6B). Among polyphenols, wedelolactone and/or 4,5-dicaffeoylquinic was abundant in all plants under different conditions. At the early stage of growth, the content of the total and individual polyphenols was mainly affected by salinity, whereas both mycorrhizal inoculation and salt stress influenced phenolic production at the later growth stage (Figures 7, 8). In detail, after 4 weeks of growth, there was a considerable effect of mycorrhizal inoculation (M) on the contents of four flavonoids (at least *P* < 0.05), five hydroxycinnamic acids (at least *P* < 0.01), and demethyl wedelolactone (*P* < 0.001). Salinity had a substantial impact on the level of all polyphenol compounds tested (at least *P* < 0.05), except demethyl wedelolactone and 5-*O*-caffeoylquinic acid. Interactions between two main effects on 3,5-dicaffeoylquinic acid (*P* < 0.001), ferulic acid (*P* < 0.01), feruloylquinic acid (*P* < 0.001), 4,5-dicaffeoylquinic acid (*P* < 0.05), and luteolin (*P* < 0.001) were found. When plants reached 8 weeks of age, mycorrhizal colonization significantly influenced all polyphenol compounds (at least *P* < 0.05), except demethyl wedelolactone. Likewise, salinity elicited sharp changes in all polyphenols (with at least *P* < 0.01). Interactions between two main effects on most polyphenols were recorded (at least *P* < 0.05, except luteolin-glucoside and demethyl wedelolactone).

After 4 weeks of growth, mycorrhizal colonization resulted in a significant increase in the total polyphenols (by 139%) in non-stress plants. Such a tendency was observed in the content of wedelolactone (105%), 3,5-dicaffeoylquinic acid (404%), 4,5-dicaffeoylquinic acid (1,281%), and feruloylquinic acid (2,901%). Moderate salinity significantly induced higher total phenolics (166%) and seven individual polyphenols such as wedelolactone (134%), ferulic acid (239%), 3,5-dicaffeoylquinic acid (842%), 4,5-dicaffeoylquinic acid (1,436%), 4-*O*-caffeoylquinic acid (336%), caffeic acid (171%), and luteolin (287%) in uncolonized plants at 4 weeks, while these increments were not found under high salt stress, except ferulic acid, 5-*O*-caffeoylquinic acid, and luteolin. By contrast, under both salt stresses, the decrement trend was seen in the content of total polyphenols and wedelolactone, luteolin-7-glucoside, and feruloylquinic acid in mycorrhizal plants, being more severe under high salt stress, whereas there were no significant changes in the concentrations of ferulic acid, 4,5-dicaffeoylquinic acid, 3,5-dicaffeoylquinic acid, quercentin-3-arabinoside, 4-*O*-caffeoylquinic acid, 5-*O*-caffeoylquinic acid, and 3,4-*O*-dicaffeoylquinic acid in colonized plants as compared with the counterparts of non-stress mycorrhizal ones. Noticeably, under moderate salinity, the concentrations of wedelolactone, ferulic acid, and 4-*O*-caffeoylquinic acid were substantially higher in non-AM plants than in AM plants. Nevertheless, the fungal symbiont markedly enhanced the content of luteolin (62.3%) in the host plants in relation to those of non-AM plants. Besides, demethyl wedelolactone was under the detection limit in colonized plants under such stress, but their feruloylquinic acid was detectable. When exposed to high salinity (200 mM NaCl), fungal colonization positively influenced the level of 3,5-dicaffeoylquinic acid (37-folds more than that of the corresponding uncolonized plants), 4,5-dicaffeoylquinic acid (17-folds), feruloylquinic acid (detectable versus undetectable), and luteolin-glucoside (detectable), but negatively affected the content of ferulic acid (decreased by 268% over the corresponding uncolonized plants) in colonized plants at 4 weeks.

After 8 weeks of growth, salinity led to a significant reduction in the content of total polyphenols and 11 phenolic compounds in non-AM plants, being more severe under high saline conditions. In 8-week mycorrhizal plants, moderate salinity also depressed the content of total polyphenols and eight phenolic substances, but the descending trend was alleviated in most bioactive compounds under high saline conditions. The level of few metabolites such as ferulic acid, 4-*O*-caffeoylquinic acid, and luteolin-glucoside was even profoundly enhanced by 93.7, 204, and 74%, respectively, in AM plants exposed to high salinity relative to non-stress AM plants. Noticeably, after 8 weeks of growth in the presence of 200 mM NaCl, the concentrations of all phenolic compounds were sharply inclined in mycorrhizal plants in relation to the counterparts in non-AM plants, except demethyl wedelolactone. The highest and lowest increases induced by AMF were 4-*O*-caffeoylquinic acid (more than 10-folds) and luteolin-glucoside (160%), respectively.

Interestingly, significant changes in the content of phenolic compounds in non-AM and AM plants were observed over time. Under non-stress conditions, there were substantial increases in the content of most polyphenols in AM (eight phenolics) and non-AM plants (10 phenolics) at 8 weeks versus their levels in the corresponding plants at 4 weeks. Considerable decreases in the content of seven individual phenolics were found in uncolonized plants treated with 100 mM NaCl 8 weeks after growth versus those 4 weeks after growth. By contrast, substantial inclines in the concentration of three polyphenols and a dramatic decrement in luteolin level (73%) were detected in colonized plants exposed to moderate salinity at 8 weeks relative to their counterparts at 4 weeks. A significant augmentation in the level of two polyphenols and remarkable declines in four phenolic concentrations were found in uncolonized plants subjected to 200 mM NaCl at 8 versus 4 weeks. Contrariwise, pronounced increases in the concentration of all phenolic compounds were recorded in colonized plants exposed to high salinity at the early stage of plant growth relative to those at the later stage.

### Principal Component Analysis of Individual Polyphenols

**FIGURE 9:**
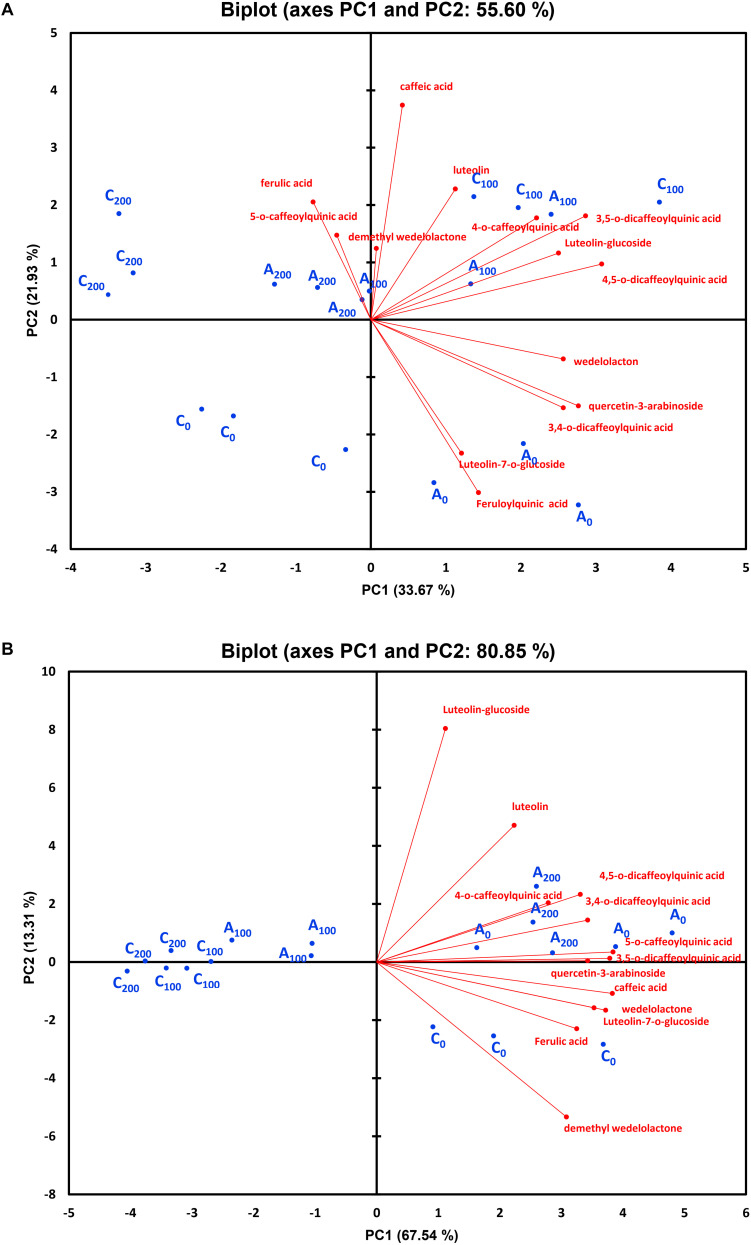
Principal component analysis of individual polyphenols in the leaves of *Eclipta prostrata* inoculated with arbuscular mycorrhiza or not inoculated under non-stress, saline conditions 4 **(A)** and 8 weeks **(B)** after inoculation. C_0_, control treatment (plants without mycorrhiza) under non-saline conditions; C_100_, control treatment exposed to 100 mM NaCl; C_200_, control treatment exposed to 200 mM NaCl; A_0_, mycorrhizal treatment under non-saline conditions; A_100_, mycorrhizal treatment exposed to 100 mM NaCl; A_200_, mycorrhizal treatment exposed to 200 mM NaCl.

Principal component analysis of individual polyphenols were performed, independently for each harvest time, to correlate variables determined under different conditions at 4 and 8 weeks. Four (at 4 weeks) and three (at 8 weeks) components showed eigenvalues higher than 1 (Supplementary Tables S1, S2). The results demonstrated that 55.6 and 80.8% of the total variation were explained by the first two principal components (PC1 and PC2) at 4 and 8 weeks, respectively (Figure 9). After 4 weeks of growth, 33.6% of the total variation was covered by the PC1, which had strong positive associations mainly with wedelolactone, 4,5-dicaffeoylquinic acid, quercetin-3-arabinoside, 4-*O*-caffeoylquinic acid, 3,4-*O*-dicaffeoylquinic acid, 3,5-*O*-dicaffeoylquinic acid, and luteolin-glucoside. PC2, covering 21.9%, was contributed primarily by caffeic acid (positive association) and feruloylquinic acid (negative association). In the next stage of plant growth (8 weeks), as much as 67.5% of the total variation was covered by the PC1, which was positively influenced by all phenolic compounds (13 individual polyphenols with luteolin and luteolin-glucoside having fewer impacts). PC2 explaining 13.3% of the total variance is positively influenced mainly by luteolin-glucoside and luteolin but negatively impacted principally by demethyl wedelolactone. At 4 weeks, high positive correlations between ferulic acid and 5-*O*-caffeoylquinic acid, demethyl wedelolactone and caffeic acid, 4-*O*-caffeoylquinic acid and 3,5-*O*-dicaffeoylquinic acid, 3,5-*O*-dicaffeoylquinic acid and luteolin-glucoside, luteolin-glucoside and 4,5-*O*-dicaffeoylquinic acid, quercetin-3-arabinoside and 3,4-*O*-dicaffeoylquinic acid, and luteolin-7-*O*-glucoside and feruloylquinic acid could be seen, whereas there were negative associations between feruloylquinic acid/luteolin-7-*O*-glucoside and ferulic acid/5-*O*-caffeoylquinic acid (Figure 9A). At 8 weeks, there were robust positive correlations between 4-*O*-caffeoylquinic acid and 4,5-*O*-dicaffeoylquinic acid, 5-*O*-caffeoylquinic acid and 3,5-*O*-dicaffeoylquinic acid, 3,5-*O*-dicaffeoylquinic acid and quercetin-3-arabinoside, and wedelolactone and luteolin-7-*O*-glucoside (Figure 9B). The biplot also demonstrated a relatively clear discrimination among the groups of the control treatment (C_0_) and mycorrhizal treatment (A_0_) under non-saline conditions, the control treatment under 200 mM NaCl (C_200_), and the other group (A_100_ + A_200_ + C_100_) at 4 weeks. Differences among C_200_, C_0_, and A_0_ groups were distinguished by PC1, while PC2 discriminated between the salinity stresses and non-saline groups at 4 weeks. Four different clusters—C_0_, A_100_ (AM inoculation under 100 mM NaCl), A_0_ + A_200_ (AM treatment under non-stress and high salt stress conditions), and C_100_ + C_200_ (control treatment in the presence of 100 and 200 mM NaCl)—were recognized at 8 weeks. Obviously, AM inoculation under non-saline and high saline conditions influencing individual polyphenols was different from the other groups at the later stage of plant growth.

## Discussion

In our preliminary experiment, although *A. lacunose* treatment had considerably lower mycorrhizal colonization than others at 4 weeks, no significant differences in AM colonization among colonized plants in different AM treatments were recognized after 8 weeks of growth. In contrast to observations in artichoke, six AMF isolates exhibited different colonization dynamics at 7, 12, and 23 weeks after inoculation in two cultivars (Avio et al., 2020). Indeed, different colonization of AMF species or isolates may reflect various AM colonization strategies, and/or AMF strains had a particular selectivity to their host plants (Duc and Posta, 2018). However, our different results might be the outcome of the specific combination of AM isolates and this medicinal plant. It is worth mentioning that root mycorrhizal colonization has been shown not necessarily to correlate with the host performances’ promotion (Duc et al., 2018). Our findings showed that the effect of AMF species on growth characteristics of host plants was different (Table 1). *S. deserticola* remarkably lowered shoot weight and plant height in relation to non-AM plants, while plant height of *A. lacunose* treatment was considerably reduced as compared with that of uncolonized plants. These growth reductions of colonized plants may result from the unbalanced trade between the host plant and AMF, i.e., the AMF–plant interaction could be parasitism (Schmidt et al., 2011) due to the carbon drainage in the host inflicted by the fungi (Fitter, 2006). Interestingly, the application of the AMF mixture considerably increased shoot weight but not other growth parameters. This may highlight that the responsiveness of the host plant varies between different inocula. Besides growth characteristics, AM treatments did not elicit any substantial changes in proline and total phenolic content during plant growth, except lower total phenolic concentration in *S. deserticola* colonized plants 8 weeks after inoculation. Hence, the AM mixture (Symbivit) was chosen for mycorrhizal inoculation in our salt stress experiment.

Although AMF have been shown to improve plant performance under salinity in many plant species (Santander et al., 2019; Ait-El-Mokhtar et al., 2020; Amanifar and Toghranegar, 2020), responses of mycorrhizal *E. prostrata* plants to salinity, especially in terms of bioactive compounds, have not been addressed. In the present work, mycorrhizal colonization rate was markedly reduced after 4 weeks of plant growth due to the high saline level (200 mM NaCl), but not at the later stage of plant growth (Figure 2). The adverse impact of high salt stress on AM colonization capacity at the early stage of plant growth could be the consequence of the direct inhibitory effect of NaCl on extraradical hyphal growth, sporulation, and spore germination (Garg and Chandel, 2015), but subsequently, AMF may adapt to such salt level. Previous reports illustrated that salinity lessened mycorrhizal colonization though it was dependent on cultivars and AMF isolates (Santander et al., 2019; Wang et al., 2019). Plants exposed to salt stresses display inhibited growth mainly as a consequence of the deleterious effects of the high osmotic potential of salt-affected soils and ionic imbalances, which disrupt normal metabolism, water, and nutrient uptake (Santander et al., 2017). Plant biomass is the most prominent and direct attribute showing symbiosis-mediated plant performance under salt stress. AM inoculation has been reported to enhance the growth characteristics of several plants subjected to saline conditions, such as lettuce (Santander et al., 2019), date palm (*Phoenix dactylifera* L.) (Ait-El-Mokhtar et al., 2020), and the medicinal plant *V. officinalis* (L.) (Amanifar and Toghranegar, 2020). These findings support that the beneficial effects of AM application on the growth of host plants under salinity are consistent with our observation (increased fresh shoot weight, leaf number, and leaf area) under moderate salinity. Several mechanisms have been proposed to explain the higher host growth under salinity, such as improved nutrient uptake, net photosynthetic rate, stomatal conductance, relative water content, and osmoprotection; enhanced antioxidative enzymes; and maintenance of ionic homeostasis (Evelin et al., 2019). Furthermore, fungal symbiosis has the ability to retain Na^+^ in the roots, probably in intraradical hyphae, which reduces its availability to the host (Giri et al., 2007; Rivero et al., 2018). A substantial decrement in Na^+^ translocation from roots to shoots was found in colonized plants under salinity (Moreira et al., 2020), which may contribute to the higher plant growth. However, our results illustrated that the host growth promotion failed to be detected under high salt conditions (200 mM NaCl), suggesting that AM benefits depended on the severity of salt stress. Thus, our findings suggest that AM application could be applied to this plant production in agricultural areas with saline irrigation below 100 mM NaCl (EC = 10 dS m^–1^) and/or slightly and moderately saline soils (EC of the saturation extract from 4 to 8 dS m^–1^).

Our findings also revealed that proline production was stimulated in AM symbiosis under moderate salt conditions 8 weeks after inoculation, in accordance with the report of Santander et al. (2019). Contrariwise, Amanifar and Toghranegar (2020) illustrated that under salt stresses, a lower proline level was recorded in the leaves of the medicinal plant *V. officinalis* inoculated by either *R. irregularis* or *F. mosseae*, which may indicate mitigation of the stress (e.g., maintaining the ratio of K^+^/Na^+^) upstream of proline synthesis (Evelin et al., 2019). A high proline level in our results may be associated with less oxidative damage in moderate-salted mycorrhizal plants. In fact, proline serves as an osmoprotectant and effective ROS scavenger, stabilizing cellular structures and membranes (Meena et al., 2019), thus decreasing ROS damage as shown by the findings in this study. Besides, proline lowers K^+^ efflux induced by Na^+^, leading to the increment in K^+^ concentration within plant cells (Hossain et al., 2014). As crucial parts of the photosynthetic apparatus, photosystems (PS) I and II are susceptible to saline conditions. Salinity can demolish the reaction center of PS II, disturb electron transport from PS II to PS I, and eventually result in a drop in photosynthesis (Wang et al., 2019). We found that the maximal photochemical efficiency of PS II (*F*_*v*_/*F*_*m*_) was not affected by salinity, indicating that salinity did not impair the photosynthetic system under our experimental conditions. Contrast observations were obtained in early investigations (Wang et al., 2019). The reasons may be attributed to differences in growth conditions, stress treatments, and stress duration, as well as specific interaction between fungal and plant partners, as reported in earlier studies (Duc et al., 2018; Amanifar and Toghranegar, 2020).

It is well known that plants can activate antioxidant systems where SOD, POD, and CAT are important enzymes to protect themselves against oxidative stress caused by ROS. SOD functions as the first defense line to deal with ROS, catalyzing the dismutation of superoxide radical (O_2_^–^) or singlet oxygen (^1^O_2_) to H_2_O_2_ and O_2_ (Mittler, 2002). H_2_O_2_ is a potentially destructive subproduct of oxygen metabolism and is scavenged from cell compartments *via* CAT and peroxidases (Mittler, 2002). Under moderate salt stress, the activity of POD and CAT at 4 weeks was highly induced in mycorrhizal plants, while mycorrhizal application did not change the activity of SOD at both times of measurement. This may suggest that at the first stage of plant growth, POD and CAT were two major antioxidative enzymes in mycorrhizal *E. prostrata* plants to alleviate oxidative stress caused by moderate saline conditions. The present results concur with those in cucumber plants (Hashem et al., 2018) and date palm plants (Ait-El-Mokhtar et al., 2020). By contrast, at the later stage, fungal symbiosis did not change the activity of these enzymes in response to salt stresses. This may imply that AM-induced defense enzymes under abiotic stresses varied with plant age (Mayer et al., 2019). It is most likely that mycorrhizal plants activated different antioxidative enzymes and/or non-enzymatic antioxidants to cope with stress 8 weeks after inoculation.

Phenolic substances, the most pronounced secondary metabolites present in plants, play a crucial role in the formation of various biomolecules protecting plants against stresses (Saxena et al., 2015). Increasing the phenolics content may contribute to osmoregulation, ROS protection, or the general defense systems of salt-stressed plants (Alqarawi et al., 2014). Boosted total phenolic level has been shown in salt-stressed *Ephedra aphylla* plants (Alqarawi et al., 2014) and *V. officinalis* plants (Amanifar and Toghranegar, 2020) due to mycorrhization. Our results indicated that total phenolic accumulation (measured by HPLC) was positively influenced by AM inoculation under non-stress (at 4 weeks) and saline conditions (at 8 weeks). Under moderate salt stress, total polyphenol content was heightened at 4 weeks but declined at 8 weeks in non-AM plants. Conversely, in moderate-salted mycorrhizal plants, total phenolics remained unchanged at the first stage of plant growth but was reduced at the later stage as compared with that of non-stress colonized plants. Still, the diminishment was alleviated in AM plants relative to non-AM plants in the presence of 100 mM NaCl at 8 weeks. This may reflect different strategies between non-AM and AM plants exposed to moderate salinity during the growth stages in the activation of phenolic production to diminish oxidative damage caused by ROS. Indeed, mycorrhizal plants effectively activated POD and CAT at 4 weeks and produced higher total phenolic content at 8 weeks than uncolonized plants to cope with oxidative stress. In the presence of 200 mM NaCl, mycorrhizal inoculation promoted higher total phenolic content in plants, particularly 8 weeks after inoculation; however, this response was not effective in detoxifying ROS in colonized plants due to lower biomass production in AM plants subjected to high salinity.

No data on the production of phenolic compounds in *E. prostrata* plants with or without AM inoculation under salinity stress have been reported. Polyphenols possess antioxidant properties, therefore not only contributing to plant defenses against oxidative stress but also promoting human health benefits for their antioxidant, anticarcinogenic, cardioprotective, antihypertensive, anti-inflammatory, anti-allergic, anti-arthritic, and antimicrobial activities (Lin et al., 2016). Natural antioxidants such as polyphenols have been intensively studied in the last few years because of restrictions on the use of synthetic antioxidants and enhanced public awareness of health-related issues (Bhuyan and Basu, 2017). In our previous study, nine major polyphenols were identified and measured in *E. prostrata* plants (Vo et al., 2019). Noticeably, in the current work, we extended identifications to 14 individual phenolics. The findings showed that wedelolactone, an important phenolic compound to prevent inflammatory diseases and cancer in humans (Sarveswaran et al., 2012), was one of the two main components of phenolic compounds in plants during growth stages under different conditions, which is in line with the earlier results (Vo et al., 2019). The difference in the second main constituent of polyphenols (4,5-dicaffeoylquinic acid in the present experiment versus demethyl wedelolactone in our previous one) may be attributable mainly to the different substrate volume we applied. The results demonstrated that under non-stress conditions, AM colonization considerably altered the content of six polyphenols in plants during growth stages, which is a confirmation with our previous findings showing that individual phenolics were influenced by AMF in this medicinal plant (Vo et al., 2019). The reasons may be due to the mechanisms underlying AMF–plant interaction during mycorrhization. AM colonization could induce a secondary metabolism response in the leaves and enhance abscisic acid biosynthesis and flavonoid and terpenoid biosynthesis regulated by jasmonate in the leaves (Adolfsson et al., 2017). Moreover, the changes in phenolic compound accumulation may be related to global metabolic alterations such as the majority of sugars, organic acids, amino acids, fatty acids, and phenolic acids in mycorrhizal shoots (Saleh et al., 2020). In our findings, higher aboveground biomass in colonized plants may result from an improved nutrient, water uptake, and photosynthesis of AM plants, leading to higher production of primary metabolites, which are the main precursors for the biosynthesis of phenolic compounds through the shikimic acid pathway (Lin et al., 2016). Therefore, changes in carbohydrate metabolism in colonized plants could alter the biosynthesis of phenolic substances (Pedone-Bonfim et al., 2018). Interestingly, the AMF-induced changes in polyphenol profiles at both stages of plant growth in this work and our previous one were not the same, probably owing to differences in plant age (4, 8, and 7 weeks) and substrate volume. We also observed that plant age markedly influenced the content of total and individual phenolic substances in AM and non-AM plants (8 versus 4 weeks). In fact, many biological factors, including developmental ones, contribute to the accumulation of secondary metabolites in plants (Broun et al., 2006). Developmental factors have an influence on the initiation and consequent differentiation of cellular structures related to secondary metabolites’ biosynthesis and storage (Broun et al., 2006). Notably, developmental stages of the plant impact the expression pattern of biosynthetic genes of secondary metabolites (Sanchita and Sharma, 2018), which could explain the changes in the content of phenolic constituents during plant growth in this study.

Salt stress stimulates phenolic compound accumulation in plants as a defense mechanism to stress (Parvaiz and Satyawati, 2008). Therefore, this abiotic stress is one of the robust elicitors of secondary metabolite production of many herbs (Bistgani et al., 2019; Behdad et al., 2020; Boughalleb et al., 2020). In this study, salinity had the trend toward increasing and remaining phenolic compounds unchanged in non-AM plants in the presence of 100 and 200 mM NaCl, respectively, at the first stage of growth but dropped them at the later stage. On the contrary, the decline in polyphenols caused by salt treatments was observed in mycorrhizal plants during the growth stage, with the mitigation at the later growth stage. Different behaviors in individual phenolics accumulation between non-AM and AM plants under saline conditions may result from the difference in the biochemical and physiological status in the host due to mycorrhization and AM benefits. On top of that, mycorrhizal inoculation caused changes in the content of many tested secondary metabolites of *E. prostrata* plants under both salinity levels at the early stage of plant growth. Noticeably, at the later growth stage, AMF enhanced all phenolic components in the host plants under high salt stress (200 mM NaCl). According to Rivero et al. (2018), in response to salt stress, various compounds with antistress properties differentially accumulated in mycorrhizal roots. The fungal symbiont also influenced the age-related changes in the leaf metabolome and partially halted senescence in the leaves, possibly resulting in better metabolite accumulation (Shtark et al., 2019). Taken altogether, these metabolic alterations induced by AMF may be the reason for the profound impact on polyphenol profiles of this medicinal plant under saline conditions during growth stages. Discrepant observations on phenolic compound accumulation in mycorrhizal plants subjected to salt stress were reported. Santander et al. (2019) pointed out considerably lower phenolics in the leaves of two lettuce cultivars colonized by AMF under salt stresses. Contrariwise, several studies have illustrated significant inclines in phenolic substances in AM plants (Hashem et al., 2018; Amanifar and Toghranegar, 2020). However, most previous studies only examined polyphenol profiles at one harvest time. Here, we observed both trends (increase and decrease) in phenolic compounds under salt stress conditions, depending on plant age and stress severity. It may be owing to the fact that the secondary metabolic pathways and their regulation are incredibly susceptible to environmental factors and growth stages since the expression of genes involved in their pathways or their encoded protein activities are changed at different plant ages and/or in the presence of various stresses (Sanchita and Sharma, 2018; Li et al., 2020).

## Conclusion

In this work, mycorrhization and co-treatment of AMF and salinity elicited significant changes in the accumulation of phenolic compounds in the medicinal plant *E. prostrata*. The findings illustrated that the positive effect of AM inoculation on polyphenol profiles was dependent on stress severity and plant growth stage. Mycorrhizal inoculation under moderate salinity showed a higher plant tolerance during plant growth, but under high saline conditions, the higher accumulated content of phenolic compounds was achieved at the later plant growth stage. Hence, mycorrhizal application individually or in combination with salinity and harvest time would be a practical approach for optimizing individual polyphenol production in this medicinal plant.

This study shows how important the selection of the right date of harvest is for obtaining the optimal composition of phenolic compounds for particular purposes (such as pharmaceutical, cosmetic industries); moreover, AMF and moderate salt stress can be used to manipulate the pattern of individual polyphenol production. Further studies should investigate other bioactive compounds in this medicinal plant colonized by AMF and/or exposure to different abiotic stresses to optimize their production.

## Data Availability Statement

The original contributions presented in the study are included in the article/Supplementary Material, further inquiries can be directed to the corresponding author/s.

## Author Contributions

ND: propagation of single AMF species for inoculants, data analysis, data presentation, writing of the original draft, revision, and editing. AV: conceptualization, experimental design, mycorrhizal inoculation, plant care, stress treatments, collecting samples, measurement of AM colonization, growth parameters, chlorophyll fluorescence, proline and total phenolics, extraction of leaf samples for enzyme measurements, sample preparation for HPLC, data curation, and data analysis. IH: measurement of AM colonization, enzymes, proline, and phenolic compounds (HPLC), extraction of leaf samples for enzyme measurements, sample preparation for HPLC, and preparation of Figure 6 (HPLC diagram). HD: measurement of phenolic compounds (HPLC), writing, revision, and editing. KP: experimental design, funding acquisition, project administration, resources, supervision, writing, revision, and editing. All authors contributed to the article and approved the submitted version.

## Conflict of Interest

The authors declare that the research was conducted in the absence of any commercial or financial relationships that could be construed as a potential conflict of interest.
